# A High-Entropy Oxide as High-Activity Electrocatalyst for Water Oxidation

**DOI:** 10.1021/acsnano.2c08096

**Published:** 2023-03-13

**Authors:** Mohana V. Kante, Moritz L. Weber, Shu Ni, Iris C. G. van den Bosch, Emma van der Minne, Lisa Heymann, Lorenz J. Falling, Nicolas Gauquelin, Martina Tsvetanova, Daniel M. Cunha, Gertjan Koster, Felix Gunkel, Slavomír Nemšák, Horst Hahn, Leonardo Velasco Estrada, Christoph Baeumer

**Affiliations:** † Institute of Nanotechnology, Karlsruhe Institute of Technology, Eggenstein-Leopoldshafen 76344, Germany; ‡ Peter Gruenberg Institute and JARA-FIT, Forschungszentrum Juelich GmbH, Juelich 52425, Germany; § Advanced Light Source, Lawrence Berkeley National Laboratory, Berkeley, California 94720, United States; ∥ MESA+ Institute for Nanotechnology, Faculty of Science and Technology, University of Twente, Enschede 7500 AE, Netherlands; ⊥ Electron Microscopy for Materials Research (EMAT), Department of Physics, University of Antwerp, Antwerpen BE-2020, Belgium; # NANOlab Center of Excellence, University of Antwerp, Antwerpen BE-2020, Belgium; ¶ Department of Physics and Astronomy, University of California Davis, Davis, California 95616, United States; △ Department of Chemical, Biological and Materials Engineering, The University of Oklahoma, Norman, Oklahoma 73019, United States; ▼ Universidad Nacional de Colombia sede de La Paz, La Paz, Cesar 202010, Colombia

**Keywords:** high-entropy oxides, water electrolysis, oxygen evolution reaction, perovskite oxide catalysts, green hydrogen, scaling reactions

## Abstract

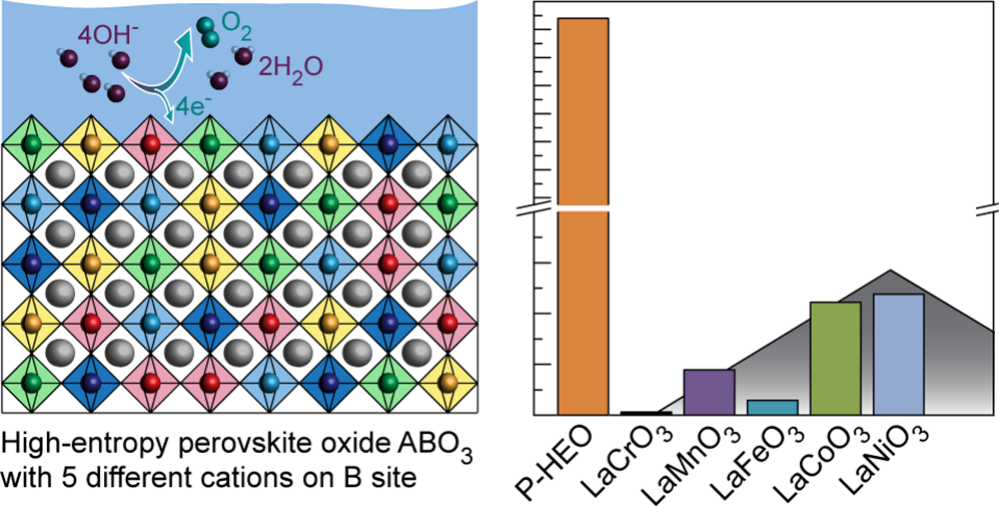

High-entropy materials are an emerging pathway in the development of high-activity (electro)catalysts because of the inherent tunability and coexistence of multiple potential active sites, which may lead to earth-abundant catalyst materials for energy-efficient electrochemical energy storage. In this report, we identify how the multication composition in high-entropy perovskite oxides (HEO) contributes to high catalytic activity for the oxygen evolution reaction (OER), i.e., the key kinetically limiting half-reaction in several electrochemical energy conversion technologies, including green hydrogen generation. We compare the activity of the (001) facet of LaCr_0.2_Mn_0.2_Fe_0.2_Co_0.2_Ni_0.2_O_3-δ_ with the parent compounds (single B-site in the ABO_3_ perovskite). While the single B-site perovskites roughly follow the expected volcano-type activity trends, the HEO clearly outperforms all of its parent compounds with 17 to 680 times higher currents at a fixed overpotential. As all samples were grown as an epitaxial layer, our results indicate an intrinsic composition–function relationship, avoiding the effects of complex geometries or unknown surface composition. In-depth X-ray photoemission studies reveal a synergistic effect of simultaneous oxidation and reduction of different transition metal cations during the adsorption of reaction intermediates. The surprisingly high OER activity demonstrates that HEOs are a highly attractive, earth-abundant material class for high-activity OER electrocatalysts, possibly allowing the activity to be fine-tuned beyond the scaling limits of mono- or bimetallic oxides.

## Introduction

Electrocatalysis is a key asset in the transition toward sustainability because it enables net-zero-carbon synthesis of value-added chemicals and chemical fuels, including power-to-X approaches.^1^ Examples include electrochemical CO_2_ reduction and water electrolysis. One of the scientifically most-studied and technologically most important reactions is the oxygen evolution reaction (OER), e.g., for green hydrogen production through water electrolysis. This complex four-step reaction suffers from exceedingly large overpotentials, necessitating research for active and stable electrocatalysts.^2^ A key limitation is the linear scaling relation between the energy of different reaction intermediates and their transition states.^3−6^ Specifically, the reaction intermediates HOO* and HO* (* denotes the active site) exhibit the same dependence on the nature of the active site.^3^ The resulting linear scaling of their adsorption energies inherently leads to an “overpotential wall” which must be overcome to maximize the OER efficiency.^7^

Perovskite-type transition metal oxides (ABO_3_) are of special interest as OER electrocatalysts because of high activities in alkaline media even without the use of platinum-group metals and their tunability of physical and electrochemical properties through substitutional doping of the A and B sites.^8−14^ This tunability is key to optimize the catalyst activity, but perovskite oxides still face the overpotential wall resulting from linear scaling.^3^ To overcome this scaling, so-called high-entropy materials were proposed as alternative OER electrocatalysts. High-entropy materials are single-phase multicomponent systems of five or more elements, typically with near-equiatomic concentrations. The multitude of available lattice sites in these materials enables rapid screening for ideal (local) composition with optimized active site properties, e.g. the relative energy levels available for catalysis.^15−17^ The close proximity of multiple possible active sites has even been suggested as a pathway to overcome scaling relations because of the different preferred adsorption sites for individual OER steps.^7,18^ The overall thermodynamic barriers of the catalytic cycle on a high-entropy material may thus be decreased through the availability of different energy levels for the individual OER steps on different adsorption sites. Accordingly, metallic high-entropy alloys have already shown great promise for the OER.^15−18^ For earth-abundant transition metal high-entropy alloys, complex oxide or hydroxide layers form at the surface, which lead to extremely low overpotentials and Tafel slopes (∼300 mV at 10 mA cm^–2^ and 29 mV dec^–1^ in 1 M KOH),^19,20^ indicating significant activity enhancement compared to standard benchmarks such as IrO_x_ or Ni/Fe layered hydroxides/oxyhydroxides.^21^

Recent advances in material synthesis^22,23^ now enable the fabrication of high-entropy oxides (HEOs), i.e., single-phase metal oxides with five or more metal cations on the same crystallographic site. Compared to high-entropy metal alloys, the presence of additional sublattices in HEOs allows dedicated engineering of physical materials properties. For example, rich magnetic properties are available through an increase in the number of microstates available to the macroscopic system,^24^ and the mixed oxygen transition metal orbital character and covalency of the employed electronic states allow further fine-tuning of the adsorption energies and charge transfer characteristics^25−27^ or even an oxygen-related active site for electrocatalysts.^28^ Accordingly, HEOs already showed high promise as battery electrodes^29^ and as (electro-)catalysts.^30−32^ The realm of HEOs was extended to perovskite oxides in 2018,^22,33^ which offer the combination of the promising properties and catalytic performance of high-entropy materials with the established high-activity platform of transition metal perovskite oxides. Despite very recent explorative accounts on HEO perovskite OER electrocatalysts,^28,34^ the electrocatalytic activity has not yet been systematically explored and separated from complex morphologies resulting from the employed synthesis methods or from the self-assembly of oxide layers at the solid/liquid interface.^19,20,34,35^ As the Jaramillo, Nørskov, Rossmeisl and Markovic groups argued already in 2011 and 2017, the comparison of intrinsic activity across multiple compositions should be performed on identical sample geometries and ideally on single crystalline surfaces.^3,36^ This approach is even more important for high-entropy electrocatalysts, where the complexity in composition, structure, and physical properties has been the main hurdle in understanding of structure–activity relationships.^37−39^

**Figure 1:**
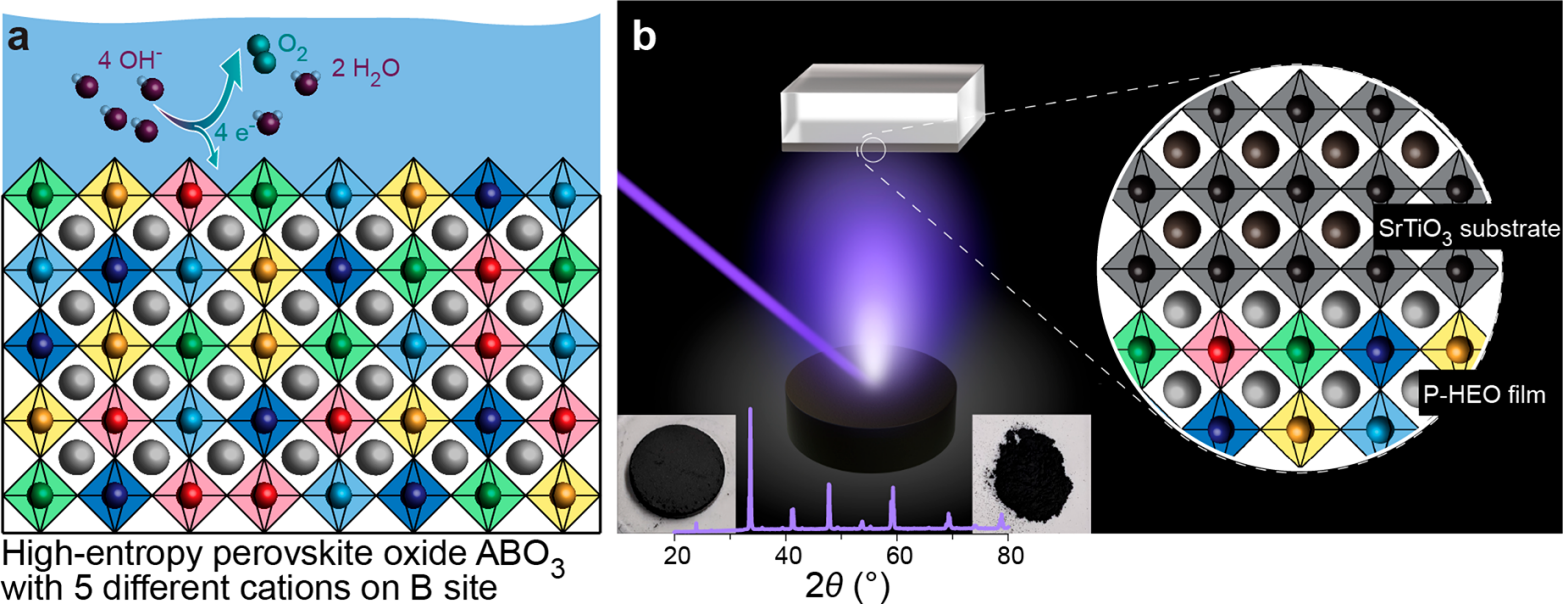
(a) Schematic illustration of the P-HEO electrocatalyst for water electrolysis. (b) The epitaxial layer is deposited via PLD. The PLD target is synthesized via sintering of P-HEO powder using reverse co-precipitation followed by calcination (see Methods section). The insets show optical micrographs of the powder and the sintered target and an X-ray diffractogram of the target, which confirm the single-phase perovskite structure, as detailed in our previous work.^22^

In this report, we explore the OER activity of the (001) facet (using the pseudocubic notation) of a perovskite oxide (P-HEO) with five B-site transition metal cations with equiatomic concentration, LaCr_0.2_Mn_0.2_Fe_0.2_Co_0.2_Ni_0.2_O_3−δ_ (Figure 1a). Cr, Mn, Fe, Co, and Ni are chosen as B-site cations because they span a wide range of binding energies in the activity volcano of the parent compounds (single B-site).^3^ This P-HEO is synthesized as an epitaxial thin film, which allows deriving structure–function relationships between the reactivity and the atomic-level surface structure and composition of a defined crystallographic facet.^9,36,40^ Using epitaxial thin films avoids the effect of complex geometries and the need for carbon-containing binders.^41,42^ We compare the P-HEO OER activity to epitaxial layers of the parent perovskite compounds and find that P-HEO exhibits drastically enhanced activity, with enhancement by a factor of 17 to 680 at fixed overpotential. These findings suggest that HEOs are a highly attractive candidate material class for high-activity OER electrocatalysts, possibly allowing fine-tuning of the activity beyond the scaling limits of mono- or bimetallic oxides, and urgently calling for mechanistic studies to investigate the origin of the activity enhancement for further electrocatalyst optimization.

## Results and Discussion

**Figure 2:**
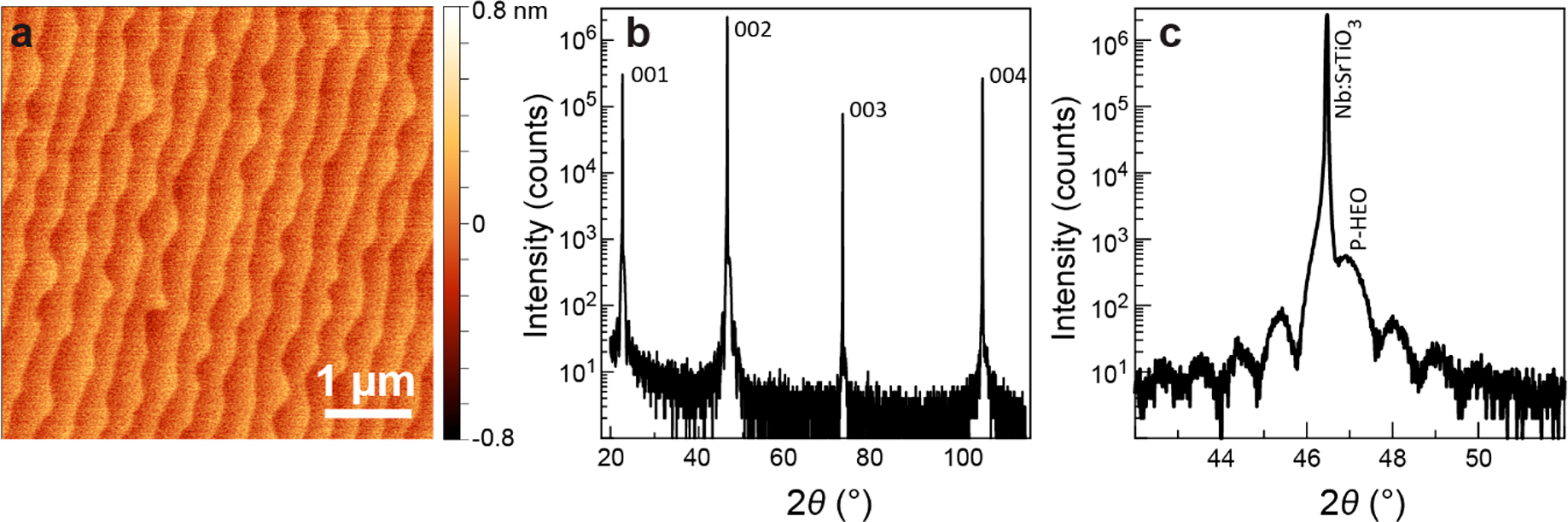
(a) Atomic force microscopy morphology of an 11 nm P-HEO deposited on a Nb:SrTiO_3_ substrate. (b,c) HRXRD patterns of the same P-HEO film, indicating epitaxial growth of the HEO thin film in the (001) orientation of the perovskite structure.

To assess the electrocatalytic activity of a well-defined, single crystallographic facet of the recently developed P-HEO, we synthesized epitaxial layers of P-HEO via pulsed laser deposition (PLD, Figure 1b) from a self-made ceramic target. The PLD parameters were chosen similar to the description in ref (24) (see experimental details). P-HEO films with an 11 nm thickness show a comparably smooth surface morphology with a root-mean-square (RMS) roughness of ∼300 pm (Figure 2a). X-ray reflectivity (XRR) confirms that the film has a thickness of ∼11 nm (supplementary Figure S1). High-resolution X-ray diffraction (HRXRD, Figure 2b,c) exhibits clear Laue fringes, indicating the high crystalline quality of the P-HEO film. The P-HEO film peaks partially overlap with the substrate peaks, and the out-of-plane lattice parameter of the film is estimated to be ∼3.86 Å. Reciprocal space mapping confirms coherent strain to the substrate (with a moderate tensile strain of −0.7%, supplementary Figure S2). X-ray photoelectron spectroscopy (XPS) with varying mean escape depth *d* revealed that the surface was slightly deficient in Ni, Co, and Fe, indicating predominant A-site termination (supplementary Figure S3).^43^ Interestingly, the 11 nm P-HEO film does not exhibit measurable in-plane electrical conductivity at room temperature (the resistance was higher than 10 MΩ), as was also observed before.^24^

**Figure 3:**
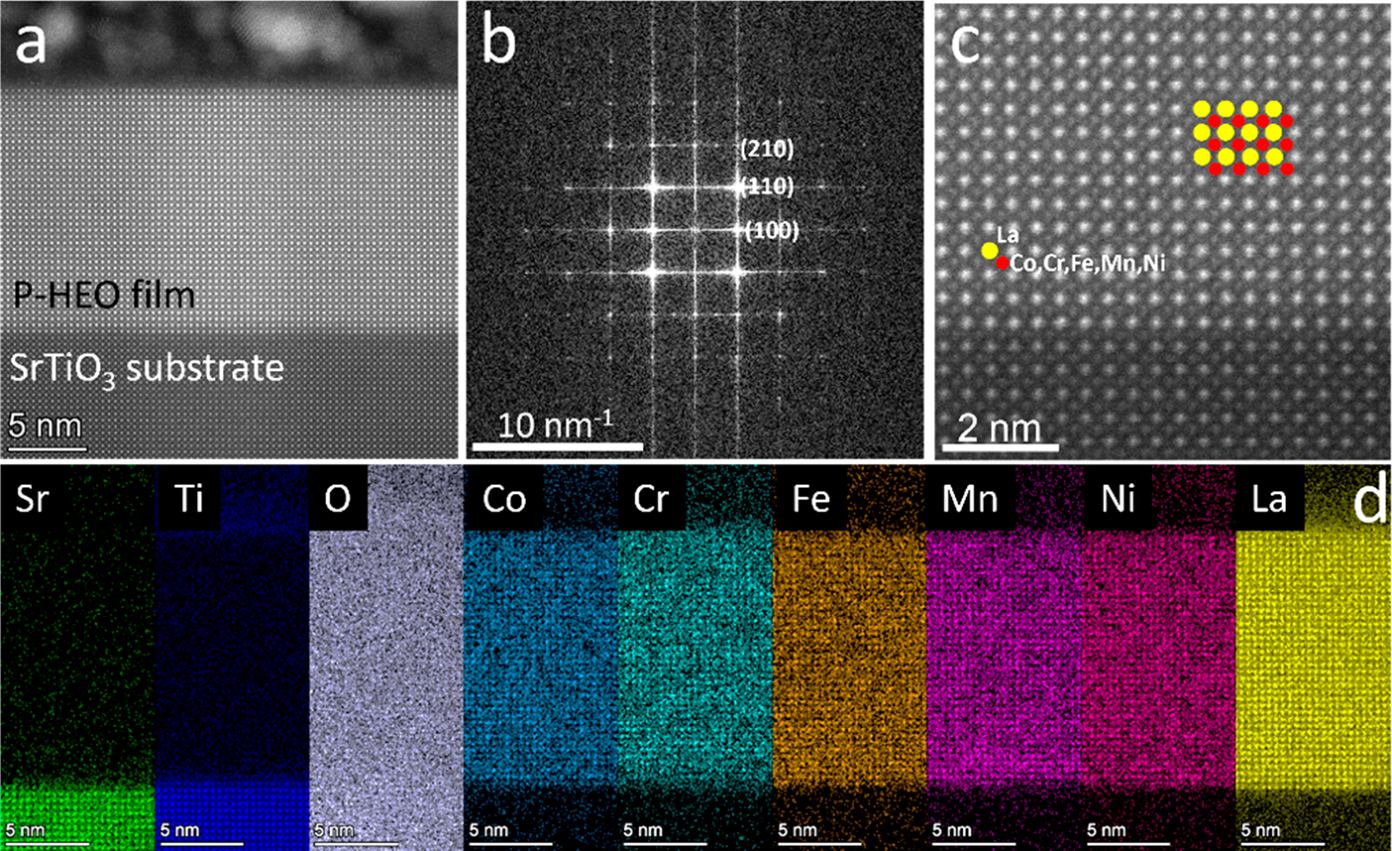
(a) STEM micrograph of a P-HEO film on an SrTiO_3_ substrate. The top surface was protected with Au/Al_2_O_3_, giving rise to clustered features above the film surface. (b) FFT of (a). (c) Magnified view of TEM micrographs of the P-HEO film. (d) EDX chemical composition maps of the film and the substrate.

Scanning transmission electron microscopy (STEM) investigation of a P-HEO thin film is shown in Figure 3, confirming the well-ordered crystal structure and coherent growth of the P-HEO layer on the substrate. The sharp interface between the substrate and the film is visible through the *Z*-contrast in high-angle annular dark-field imaging conditions, and the film shows an epitaxial relation to the substrate (Figure 3a). The fast Fourier transform (FFT) of Figure 3a, which includes both the film and the substrate, exhibits a spot pattern (Figure 3b) that lies on the ⟨100⟩ zone axis, and the high-resolution STEM image in Figure 3c confirms the well-ordered crystal structure.

An energy-dispersive X-ray spectroscopy (EDX) chemical composition map containing the substrate and film is displayed in Figure 3d. It is evident that the interface between the substrate and the film is sharp (limited intermixing). The film composition is homogeneous throughout the entire film thickness, and there is no significant segregation of specific elements at the nanoscale. The chemical composition mapping of the film (Table 1) shows that all of the transition metals are in near-equiatomic composition, confirming near-stoichiometric transfer from the PLD target to the thin film. The mapping was repeated in several locations, confirming the homogeneous composition. To summarize, our observations with HRXRD, XRR, and STEM suggest that the film has a single phase with epitaxial growth.

**Table 1:** Atomic Fraction from EDX, Position of the Energy Edge, Intensity Ratio of the Edges (*I*_*L*_3__/*I*_*L*_2__ or *I*_*M*_5__/*I*_*M*_4__), and Oxidation States of Corresponding Elements in the System, as Estimated from the Edge Position and Intensity Ratios

element	atomic fraction from EDX (%)	position of *L*_3_ EELS edge (eV)	intensity ratio	oxidation state from EELS
Cr	9.81 ± 0.8	577.3	1.8	+3^44^
Mn	11.25 ± 1.6	641.4	2.6	2+/3+ mixture^45,46^
Fe	11.1 ± 1.5	707.8	4.6	+3^46^
Co	11.1 ± 1.4	778.9	3.9	2+/3+ mixture^45^
Ni	10.3 ± 1.5			
La	46.3 ± 3	834.6	0.99	+3

Electron energy loss spectroscopy (EELS) was used to analyze the oxidation states of the constituent elements by examining the edge position and ratio of the *L*_2_- and *L*_3_-edges of Cr, Mn, Fe, and Co and the ratio of the *M*_4_- and *M*_5_-edges for La (see supplementary Figure S4 for the EELS data). The intensity ratios (*I*_*L*_3__/*I*_*L*_2__ or *I*_*M*_5__/*I*_*M*_4__) indicate that the oxidation state of Cr and Fe is +3,^44,45^ while Mn exhibits a slightly reduced +3/+2 valence,^45,46^ and Co shows stronger reduction toward +2 valence. These oxidation states are in agreement with the expected charge transfer and preferred valence states in the first row of transition metals.^47,48^ Lanthanum only exhibits +3, as expected from the stable inert gas La^3+^ configuration. The Ni *L*-edge at 850 eV is not interpretable due to the strong overlap with the La *M*_4_-edge.^49^ The STEM/EELS analysis confirms that our P-HEO has the desired perovskite phase, in contrast to previous reports that exhibited a pronounced Cr deficiency.^34^ The slight reduction of Mn and Co (and possibly of Ni) may be the result of a small oxygen deficiency due to favorable reduction enthalpy, as is frequently observed in the single B-site perovskites.^26^

To compare the OER activity of the epitaxial P-HEO with its single B-site parent compounds, (001)-oriented PLD films of LaCrO_3_, LaMnO_3_, LaFeO_3_, LaCoO_3_, and LaNiO_3_ were grown by PLD. The films of all compositions showed two-dimensional growth, resulting in a smooth surface with RMS roughness below 300 pm (supplementary Figures S5–S9). All films exhibited predominant A-site termination, as revealed by XPS analysis. While we recently demonstrated that the surface termination can have a large effect on the OER activity,^12^ this observation allows a direct comparison of the OER activity as a function of film composition. With these epitaxial, single-phase and well-ordered (100)-oriented thin films, we are thus well-positioned to systematically assess the intrinsic OER activity of the P-HEO.

**Figure 4:**
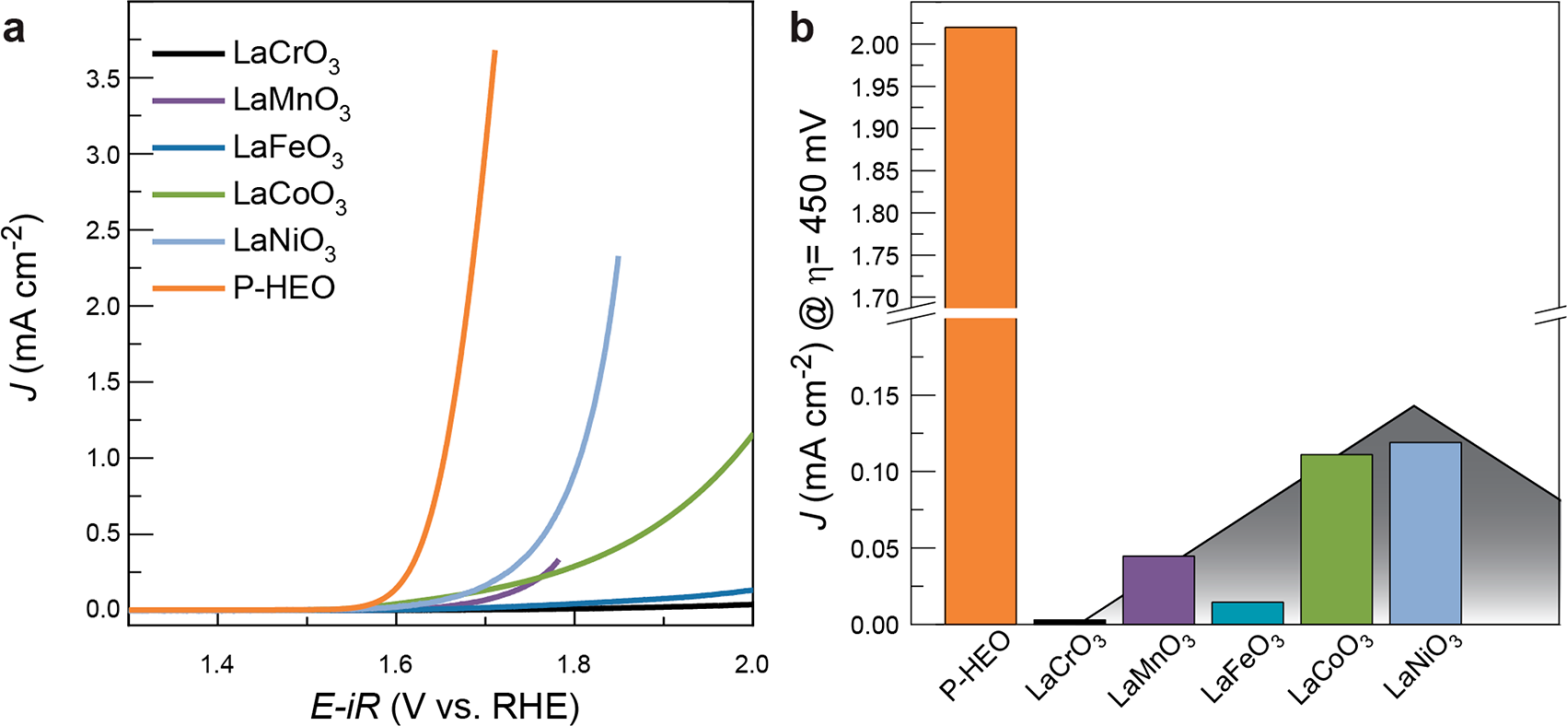
(a) Cyclic voltammetry of P-HEO and its parent compounds. The plots show the average of anodic and cathodic scans in the second consecutive cycle. (b) Comparison of the specific OER activities (current density at an overpotential of 450 mV, i.e., 1.68 V vs RHE). The underlying volcano is a guide for theeye, inspired by the predicted activity volcano from ref (50).

OER activity was measured using cyclic voltammetry (CV) in an O_2_-saturated 0.1 M potassium hydroxide (KOH) solution in a three-electrode configuration using a specially designed rotation disk electrode (RDE) setup for epitaxial thin films.^12^ Electrical contact was ensured through a sputtered Pt contact on the film edges and through a highly conductive Nb:SrTiO_3_ substrate. Electrochemical impedance spectroscopy (supplementary Figure S10) revealed that the resulting uncompensated resistance was 48.4 Ω, i.e., the electrolyte resistance in our setup, indicating that the high in-plane resistance of the P-HEO film did not limit the performance in the electrochemical cell, presumably due to sufficient out-of-plane conductivity and the integration on a highly conductive substrate. Figure 4a shows the average of the anodic and cathodic scans for the films with different compositions, indicating that P-HEO possesses an OER activity higher than that of all single B-site parent compounds. The single B-site perovskite oxides roughly follow the volcano-shaped trend predicted from density functional theory (DFT) (Figure 4b),^50^ which results from increasing energy difference between O* and HO* intermediates with increasing electronegativity of the transition metal,^3^ with the exception of LaFeO_3_, which showed a lower activity than expected based on this trend. The unexpected low activity LaFeO_3_ is associated with the insufficient electronic conductivity (visible from valence band (VB) spectroscopy, indicating low density of states at the Fermi level) and decreased hybridization between the O 2p and TM 3d bands, which will be further discussed below. The P-HEO activity is approximately 17 times higher than for LaNiO_3_ and LaCoO_3_, approximately 45 times higher than for LaMnO_3_ and more than 680 times higher than for LaCrO_3_ at the reference overpotential of 450 mV. Tafel analysis of the CV data shows a Tafel slope of 51 mV/dec for the P-HEO, confirming the improved activity when comparing to the 128 mV/dec slope observed for LaNiO_3_ (supplementary Figure S11) We note, however, that the OER mechanism cannot be solely obtained from the Tafel slope.^51^ Preliminary chronopotentiometry results (supplementary Figure S12) indicate that the activity of P-HEO remains higher than that for the parent compounds. The P-HEO lifetime also appears to be enhanced, indicating that our P-HEOs exhibit sufficient stability to compare intrinsic OER activities. But the effect of entropy stabilization for the electrocatalyst stability remains a topic for closer examination in future studies.

Similar activity trends were also observed in a previous study, which compared samples with high surface roughness and unknown surface composition and orientation.^34^ This further verifies our observation of substantial activity increase, while our results with epitaxial thin films have the distinct advantage of confirming intrinsic material trends. The surface area for all of our films is well represented with the geometric surface area because of the negligible surface roughness. The tensile strain state, which can have an impact on OER activity,^52^ and crystalline quality of the P-HEO film is also similar to the more active parent compounds LaNiO_3_ and LaCoO_3_ thin films. If we assume only one active site, lower rather than higher activity would be expected for P-HEO because the presumed active sites (i.e., one of the transition metal ions with 3+ or mixed 2+/3+ oxidation state) are also present in the parent compounds but with a higher concentration.

**Figure 5:**
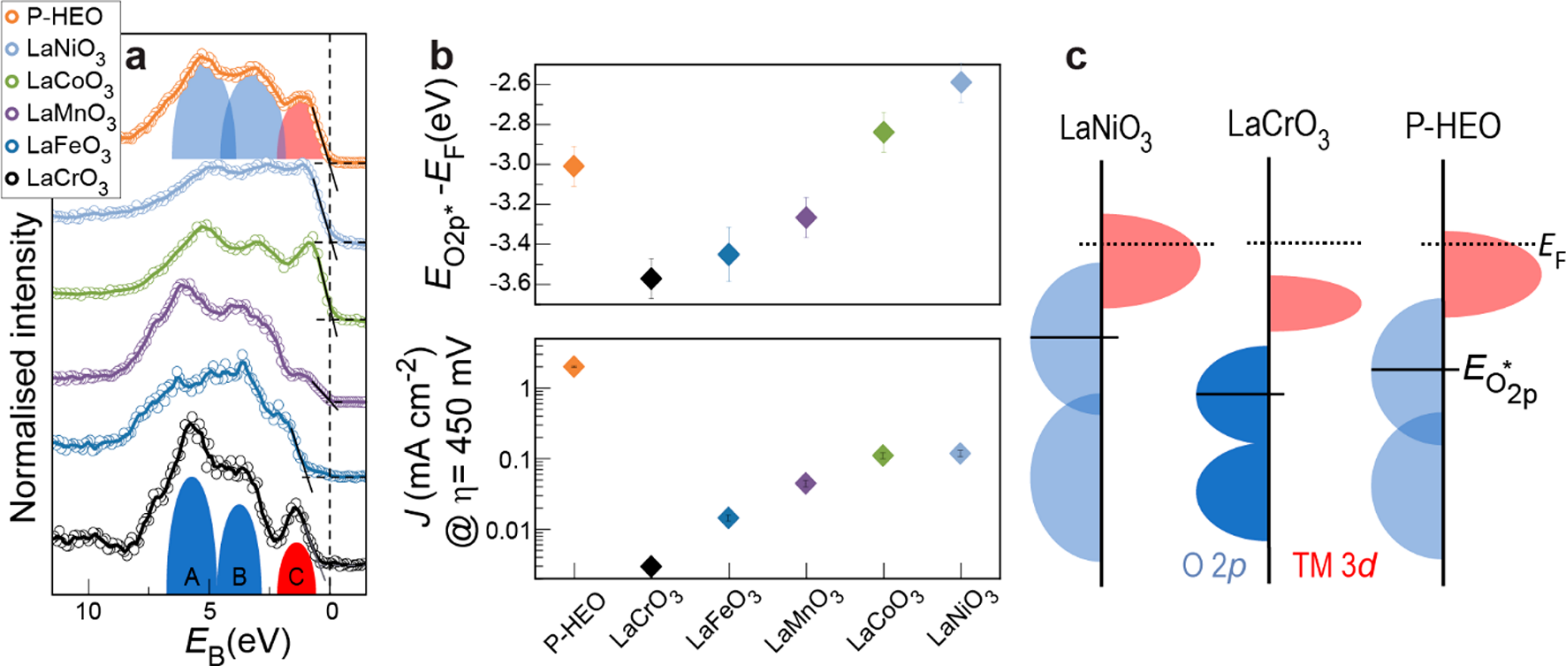
(a) Valence band spectra of P-HEO and its parent perovskite oxides. The intensity was normalized to the area of the binding energy region −2 to 8 eV. O 2p states (peaks A and B, blue) and TM 3d states (peak C, red) are indicated schematically for the examples of LaCrO_3_ (low degree of covalence) and P-HEO (high degree of covalence). Raw data are shown as open dots; lines are a guide for the eye obtained by 5-point adjacent average smoothing. The valence band maximum is indicated via the zero photoemission intensity intercept of a linear regression fit of the low-binding-energy edge of the valence band spectra. (b) O 2p nonbonding state binding energy  and OER activity for P-HEO and parent perovskite oxides. The error bars represent the possible maximum deviation of the consecutive measurements (top panel) or estimated relative errors from triplicate CV measurements (bottom panel). Note that the parent compounds are ordered by  rather than atomic number of the TM. (c) Schematic energy band diagram for LaNiO_3_, LaCrO_3_ and P-HEO. The Fermi level (*E*_F_) is labeled by dashed lines in (a) and (c), and the valence band maximum is schematically shown as linear extrapolation of the leading edge of the VB.

To clarify the possible origins of the surprisingly enhanced activity, we examine the electronic structure of P-HEO in more detail using valence band spectroscopy (Figure 5a–c). The binding energy was calibrated using the C 1s peak, and only small errors (<0.2 eV) are expected because charging is negligible in such thin films on highly conductive substrates. The valence band structures of the parent compounds are similar to previous results obtained on perovskite powders,^53^ consisting of bonding O 2p states (peak A in Figure 5a,c), nonbonding O 2p states (peak B), and TM 3d states (peak C). In previous studies, the measured charge transfer energy (the difference in energy between the occupied O 2p band center and unoccupied TM 3d orbitals)^27^ or the computed O 2p band center^54^ was used as activity descriptors for perovskite-type OER electrocatalysts. To assess the O 2p band position experimentally, we fitted the valence band spectra using Voigt functions after Shirley background subtraction (supplementary Figure S13). Our parent perovskite oxides LaCrO_3_, LaMnO_3_, LaFeO_3_, LaCoO_3_, and LaNiO_3_ exhibit a direct correlation between the measured peak position of the nonbonding O 2p states (, with respect to the Fermi level) and the OER activity (Figure 5b and supplementary Figure S14). Among the parent compounds, LaNiO_3_ is the most active for the OER and exhibits the highest , as well as the smallest energy difference between O 2p and occupied TM 3d states. Together with a broadening of the O 2p bands, this results in a higher overlap of the O 2p and TM 3d bands, corresponding to a higher covalency of the TM–O bonds. It is believed that the TM 3d–O 2p covalency and large total bandwidth are the reasons for high OER activity.^11,25^ In contrast, LaCrO_3_ shows the lowest , a large energy separation and thus a small overlap of O 2p and TM 3d bands, indicating the lowest covalency in this series. In addition, LaCrO_3_ (LaMnO_3_, LaFeO_3_) shows negligible (small) density of states near the Fermi level, indicating that insufficient electronic conductivity may further contribute to the low OER activity. The valence band structure data can also explain why LaFeO_3_ was less active compared to the expected trend (Figure 4b). The increase in exchange stability in the 3d^5^ configuration of LaFeO_3_ leads to an increase in the charge transfer gap and decrease in hybridization compared to the trend of 3d TM perovskites.^53^ Accordingly, we recover a monotonic trend when plotting the OER activity as a function of  (Figure 5b).

**Figure 6:**
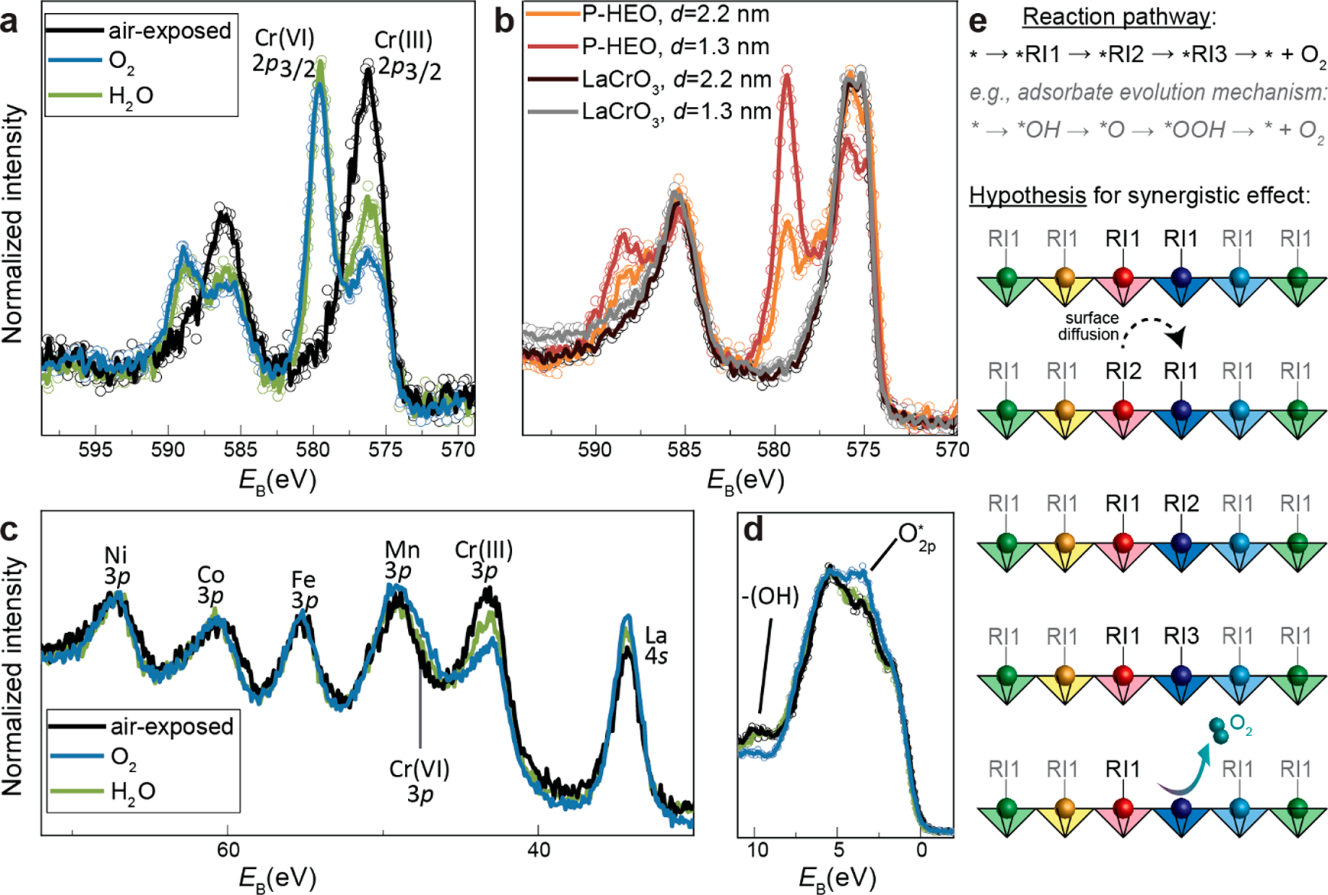
(a) Cr 2p core level of P-HEO during APXPS experiments. (b) Cr 2p core level of P-HEO with different mean escape depths *d*. Cr 2p core level of LaCrO_3_ is shown for reference. Spectra collected in UHV after annealing in O_2_. (c) TM 3p core levels of P-HEO during APXPS experiments. (d) VB spectra of P-HEO during APXPS experiments. Raw data in (a), (b), and (d) are shown as open dots; lines are a guide for the eye obtained by 5-point adjacent average smoothing. (e) Hypothetical reaction mechanism involving three reaction intermediates (RI) and surface diffusion between a strongly binding site (red) and a weakly binding site (blue).

For P-HEO, we found a valence band signature that resembles the highly covalent parent compounds. The P-HEO has a large bandwidth and TM 3d–O 2p hybridization (Figure 5a). Figure 5c shows a schematic illustration of the valence band contribution of the TM 3d and O 2p states. The binding energy for nonbonding O 2p states is lower than for LaCoO_3_ and LaNiO_3_ but higher than for LaCrO_3_, LaMnO_3_, and LaFeO_3_ (Figure 5b). Despite the high in-plane electronic resistance measured in the van der Pauw geometry, the P-HEO exhibits a substantial density of states near the Fermi level, which was previously shown to be sufficient for high-activity perovskite OER catalysts.^11^ Based on typical OER descriptors, the valence band structure of P-HEO thus suggests mediocre OER activity (lower activity than LaNiO_3_, lower or on par with LaCoO_3_). Therefore, the experimentally observed substantially increased activity compared to LaNiO_3_ shows that neither electronic structure descriptors nor simplistic arguments regarding the availability of active sites can explain the OER activity of this P-HEO. Instead, active sites and local, partial reactions of the four-step OER must be considered in a holistic or even synergistic picture, lending support to previous arguments that the distinctive arrangement of the surroundings of a given binding site tunes the electronic properties of this site.^15,55^ Two different neighboring active sites may further synergize for subsequent steps in the catalytic cycle and therefore “stabilize OER intermediates that are unfavorable” on single sites.^56^ DFT computation has shown that theoretical overpotentials may decrease in the presence of two or more transition metal sites. In this “relay race”, reaction intermediates may be passed on from weak-binding Ni and Co sites toward strong-binding Cr, Mn, or Fe sites or even to bridging O-sites.^57,58^ Such a scenario might be particularly important when considering decoupled electron–proton transfer OER mechanisms.^59,60^ It has also been discussed in other multication oxide electrocatalysts,^61^ and time-resolved spectroscopy points toward extremely fast surface diffusion of reaction intermediates that may enable this mechanism.^62^ To illustrate the possible synergistic scenario of OER catalysis at the multication P-HEO surface, we include a hypothetical reaction pathway in Figure 6. Under reaction conditions, the surface is likely hydroxylated, which we take as the reaction intermediate RI1. The first additional oxidation step may favorably occur on a strongly binding site such as Mn or Cr.^3^ Surface diffusion induces hopping to a less strongly binding reaction site, such as Co or Ni, where additional steps can occur with smaller potential steps,^3^ decreasing the overall overpotential. We note that the exact reaction mechanism for high-entropy oxides is not yet identified and needs further investigation, implying that the proposed reaction pathway remains hypothetical and could instead also involve lattice oxygen or the effect of the spin states in different TM sites.

To shed light on the role of the different cations in the P-HEO for the binding of hydroxyl groups, a key step for all suggested OER in alkaline media, we employed ambient pressure XPS (APXPS) analysis.^59,63^ For in situ measurements, the samples were cleaned by 300 °C annealing in O_2_ before exposure to H_2_O at 25 °C. The cleaned surface represents the as-prepared state (not exposed to air) because the synthesis ends with annealing and cooling in O_2_. After the annealing, the Cr 2p and Cr 3p levels are indicative of a large Cr^6+^ contribution, while after air exposure, we only find Cr^3+^ (Figure 6a,c). Measuring the same sample with different mean escape depths after annealing (Figure 6b) confirmed that the oxidation is confined to the top surface of the P-HEO film. We also note that such surface oxidation does not occur for LaCrO_3_ films (Figure 6b) unless they are treated with ozone or oxygen plasma.^64^ These results thus indicate that the oxidation of Cr is more facile in the P-HEO than in the parent perovskite. The oxidation of Cr is accompanied by an oxidation in Ni and Co as is qualitatively evident from the peak shift and low-binding energy shoulder in the 3p core levels,^11^ while the Mn and Fe valence states remain unchanged within experimental resolution (Figure 6c). Judging from previous TM 3p measurements,^11^ the average Ni valence increases by ∼0.1, and the average Co valence increases by ∼0.3 during Cr oxidation. The simultaneous Cr and Co oxidation is also confirmed by X-ray absorption spectroscopy (XAS) experiments under the same conditions (supplementary Figures 15,16), indicating that binding of surface species (including OER reaction intermediates) results in a collective response from the different TM cations. The charge transfer direction still follows the expected trend, with reduction for the more electronegative transition metals.^47^ The Ni valence change is barely visible within our experimental XPS resolution and cannot be tracked by soft X-ray XAS because the Ni *L*-edge overlaps with the La *M*-edge, which is much higher in intensity. The Ni valence change will thus not be discussed in more detail because the changes in Co valence are more pronounced.

When the O_2_-annealed surface is exposed to H_2_O vapor in the APXPS experiment, the changes in the oxidation state are partially reversed: Cr is reduced (Figure 6a,c). A Co oxidation is evident in the XAS signature (supplementary Figure S16), while no large change is appreciable at the Co 3p XPS core level. The Mn and Fe valence states remain unchanged within experimental uncertainty. All spectral changes are smaller compared to air-exposure, which can be rationalized by the low  in the APXPS experiment and the absence of other species that may bind to the surface-like carbonate groups. It was previously observed that oxide OER electrocatalysts benefit from the presence of Cr^6+^ ions, but their role in the OER is not yet fully understood.^65^ For P-HEO, the Cr (and to some degree Co) valence changes are directly connected to the adsorption of HO* and these cations have a strong tendency for facile changes in the oxidation state. The adsorption of HO* is also evident in the valence band spectra of P-HEO, where exposure to both ambient and water vapor led to the development of a clear −OH peak and a decrease in intensity of nonbonding O 2p states (Figure 6d), further highlighting the importance . The preferential valence change of Cr alongside much smaller changes in other TM oxidation states may indicate a synergistic effect on the binding of surface adsorbates, a possible reason for enhanced OER activity. One may speculate that the Cr and Co cations play an integral role in the OER mechanism in this class of P-HEO electrocatalysts and that the presence of easy-to-oxidize Cr in the HEO matrix contributes to high OER activity. The presence of multiple cations with different electronegativity and ease of oxidation apparently facilitates both the initial bonding of HO* and the following oxidation steps, which would present a deviation from the often observed scaling relations. In the framework of the Sabatier principle (optimum activity if reaction intermediates bind neither too weakly nor too strongly), one may speculate that Cr active sites may have a low thermodynamic barrier for the initial oxidation (HO* to O* in the adsorbate evolution mechanism), while Ni and Co have a lower thermodynamic barrier for additional oxidation steps (e.g., O* to HOO*), leading to a synergistic effect of adjacent TM sites of the P-HEO, as schematically indicated in Figure 6.^3^

## Conclusions

In conclusion, our findings reveal that the (001) facet of the P-HEO LaCr_0.2_Mn_0.2_Fe_0.2_Co_0.2_Ni_0.2_O_3-δ_ is highly active for catalyzing the OER. Our study provided a one-to-one comparison of the same crystallographic facet with close to identical morphology and surface termination for a range of perovskite oxides. While the single B-site perovskites follow the OER trend expected based on the binding energy of nonbonding O 2p states, in reasonable agreement with DFT-predicted activity trends, the P-HEO electrocatalyst clearly outperforms all of its single B parent compounds. Our valence band analysis showed that the electronic structure of P-HEO is of a highly covalent nature, which is beneficial for OER activity. But the activity of P-HEO is higher than expected from electronic structure considerations. APXPS analysis showed that on adsorption of reaction intermediates several transition metals exhibit a valence change, indicating a synergistic effect in the binding of surface adsorbates, which may be the cause of the enhanced OER activity. These findings necessitate detailed future clarification of the OER on P-HEO materials and possible synergistic roles of multiple active sites to enable further electrocatalyst optimization based on rational design rules and advanced materials synthesis as well as dedicated compositional screening for optimizing the activity enhancement and possible stability differences under reaction conditions. Furthermore, we note that the magnetic moment of the active sites may play a crucial role in the formation of the oxygen molecule, which possesses a triplet ground state.^66^ This will be an important point of consideration for the future investigation of high-entropy oxygen electrocatalysts. Our conclusions provide a stimulus in the field of HEOs to explore and exploit high-entropy electrocatalysts for fundamental research as well as in application. In this sense, high-entropy oxides provide a surprisingly active addition to the recent shift toward high-entropy OER electrocatalysts. Identification of ideal compositions and surface arrangement using epitaxial thin films allows one to compare intrinsic activity trends. These can, in turn, be exploited by translating thin films with optimum performance toward high-surface-area materials.

## Methods

### Thin Film Preparation

Thin films were deposited on (001)-oriented, TiO_2_-terminated, and step-terraced Nb:SrTiO_3_ substrates (0.5 wt %, etched with buffered hydrofluoric acid, followed by annealing in oxygen at 950 °C for 2 h) via PLD. The P-HEO PLD target was synthesized via sintering of perovskite powder using reverse co-precipitation followed by a calcination process. The powders were annealed at 1200 °C for 7 h, followed by uniaxial pressing at 300 MPa and a heat treatment at 1500 °C for 12 h.^67^ The P-HEO films of 12 and 16 nm were deposited with a laser fluence of 1.8 J/cm^2^ and a frequency of 10 Hz at an oxygen pressure of 0.04 mbar. The temperature of the substrate was kept at 650 °C during deposition. The distance between sample and target was 5 cm, and the laser spot size was 1.75 mm^2^. After deposition, the sample was cooled to room temperature in 0.04 mbar of O_2_.^68^

For single B-site thin films, a spot size of 1.92 mm^2^ and polycrystalline targets of desired composition were used. The optimized growth parameters per material (substrate temperature, laser repletion rate, spot size, laser fluence, substrate–target distance, O_2_ background pressure, and obtained growth rate) are summarized in supplementary Figures S5–S9.

For the TEM investigation, a Au/Al_2_O_3_/P-HEO heterostructure was fabricated on a SrTiO_3_ substrate. The Al_2_O_3_ layer was deposited at room temperature via PLD to prevent potential damage to the P-HEO thin film during lamella preparation. The Au layer was deposited via sputtering at room temperature to increase the conductivity of the sample prior to FIB lamella preparation.

### Thin Film Characterization

Surface morphology was investigated by using atomic force microscopy (AFM), with a Bruker Dimension ICON (USA). Roughness was determined as the RMS roughness of a line profile along a step edge.

XRR and HRXRD were conducted on a Bruker AXS D-8 X-ray diffractometer with a monochromated Cu source to characterize the crystallinity of the films. Additional XRD measurements were performed on a Panalytical X’pert Pro MRD with a nonmonochromated Cu source, using a nickel filter to remove the Kβ emission.

XPS measurements were conducted with an Omicron XM 1000 MkII Al Kα monochromated X-ray source and an Omicron EA 125 energy analyzer. The mean escape depth *d* is defined through the inelastic mean free path of photoelectrons λ = 2.2 nm (calculated via QUASES-IMFP-TPP2M)^69^ and the photoemission angle θ through *d* = λ cos θ.^70^ The photoemission angles used are 0 and 55°. This isotropic approach uses the straight-line approximation,^71^ as is suitable in the absence of well-characterized, material-specific angle-dependent effective attenuation lengths. For a full description of the information depth in photoemission, the readers are referred to refs (70−72). To calculate the surface stoichiometry from the measured intensities, we compared the relative intensities of the A-site and B-site peaks as a function of *d*. The stoichiometry was determined based on the integrated raw peak areas after subtraction of a Shirley background using CasaXPS. For analysis of the valence band, the binding energy was calibrated by shifting the C 1s peak of the adventitious carbon layer to 284.8 eV.

A Helios 5 dual beam (Thermo Fisher) was used for the focused ion beam process to extract cross-sectional lamellas from a 16 nm P-HEO film for transmission electron microscopy characterization. Lamella are cut at 30 kV, followed by thinning alternatively between the front and back sides at 5, 2, and 1 kV. Beam overlap was reduced to 50% to avoid potential sample bending. The thinning process was performed for a maximum of 15 s on each side. TEM characterization was conducted in a Thermo Scientific Spectra 300 TEM with acceleration voltage of 300 kV, dwell time of 2.0 μs, and a convergence angle of 21 mrad. A Super-X G2 EDS detector was used to analyze the chemical composition of the films (20 μs dwell time, beam current of 100 pA). EELS data were collected using a Gatan GIF Continuum 970 high-resolution camera in a Thermo Scientific Spectra 300 TEM with a collection angle of 100 mrad, a beam current of 80 pA to reduce damage, and a dispersion of 0.05 eV/pixel. Separate acquisitions were performed in dual EELS mode (low-energy region containing the zero loss peak for determination of the absolute energy of the different edges).

Electrical resistivity was measured in van der Pauw geometry after sputtering of gold contacts in all 4 corners of the sample. Additional testing was performed by connecting the gold contacts to a multimeter.

### Electrochemical Characterization

To perform electrochemical experiments with epitaxial thin films on 10 × 10 × 0.5 mm^3^ single crystal substrates, we used a custom-made adapter to press the sample back side to the Pt plug of a rotating disk electrode (RDE, Pine Research). 50 nm Pt connections from the sample back side to the front side ensured electrical contact with the Nb:SrTiO_3_ substrate and the epitaxial layers. On the front side, a film area of 7.5 mm diameter was exposed to the electrolyte and sealed using an O-ring (Kalrez, ERIKS, Germany). The RDE shaft was rotating at 1600 rpm. Electrochemical testing was performed using a Parstat 4000 potentiostat (cyclic voltammetry sweep rate 10 mV/s), in a 150 mL alkaline-resistant Teflon cell (Pine Research) with a Pt wire as a counter electrode. Electrochemical impedance spectroscopy was conducted with the amplitude of 10 mV at open-circuit potential and the correction for the cell resistance (IR correction, typically 45–55 Ω) was based on the high-frequency intercept of the real impedance. The electrolyte solution of 0.1 M KOH, prepared by dissolving KOH pellets (Sigma-Aldrich, 99.99%) in deionized water. The electrolyte was O_2_-saturated prior to testing for at least 30 min and maintained under an O_2_ atmosphere during testing. All electrochemical measurements were performed at room temperature, following the recommended practices for comparison and benchmarking of the model electrocatalyst systems.^73^ Potentials were referenced to a Hg/HgO reference electrode (C3 Prozess-und Analysentechnik, Germany), which was periodically calibrated to the reversible hydrogen electrode (HydroFlex, USA) in 0.1 M KOH with typical values of ∼890 mV. All of the OER testing was performed on a fresh electrode that had not undergone previous testing. Cyclic voltammetry was first performed in the pseudocapacitive redox phase change region (∼0.9 to 1.75 V vs RHE) at scan rates between 10 and 500 mV s^–1^, followed by OER testing performed from 0.9 to 1.9 V vs Hg/HgO at a scan rate of 10 mV s^–1^. For LaMnO_3_, unstable behavior was observed for potentials exceeding 1.75 V vs RHE, so only data below 1.73 V vs RHE was reported. The CV data were capacitance corrected through averaging the forward and backward scans. The second cycle is shown for each sample.

### Ambient Pressure XPS

Ambient pressure X-ray photoelectron spectroscopy was performed using the soft X-ray beamline 9.3.2 at the Advanced Light Source, Lawrence Berkeley National Laboratory with a photon energy of 750 eV. The measurements were carried out in the as-received state of the thin film at UHV conditions as well as under exposure of the sample with *p*(O_2_) = 75 mTorr at *T* = 300 °C and, subsequently, at *p*(H_2_O) = 75 mTorr at room temperature. The H_2_O reservoir was prepared from deionized water (Millipore, >18.2 MΩ cm) and degassed by three consecutive freeze–pump–thaw cycles. For each sample state, a full set of La 4d, Cr 2p, Mn 2p, C 1s, and O 1s as well as the shallow core level region including the valence band was recorded. The respective partial pressures of the O_2_ and H_2_O atmosphere were adjusted at room temperature, and the measurements were started approximately 20 min after the probing conditions have stabilized (and the C 1s contribution has vanished upon O_2_ annealing). The heating rate in an O_2_ environment was ∼50 °C/min, while the cooling rate was ∼10 °C/min. Evaluation of the XPS spectra was performed using KolXPD.

## Supporting Information Available

Additional XPS, XAS, XRD, AFM, and electrochemical analysis (PDF)

## Notes

Kante, M. V.; Cunha, D. M.; Weber, M. L.; van den Bosch, I. C.G.; Heymann, L.; van der Minne, E.; Ni, S.; Falling, L. J.; Koster, G.; Gunkel, F.; Nemšák, S.; Hahn, H.; Velasco, L.; Baeumer, C. A High Entropy Oxide as High-Activity Electrocatalyst for Water Oxidation. **2022**, 2x0v7. *ChemRxiv*; 10.26434/chemrxiv-2022-2x0v7 (accessed Feb 18, 2023).
