# Supplementary material for: A High-Entropy Oxide as High-Activity Electrocatalyst for Water Oxidation
Source: ACS Nano. 2023 Mar 13;17(6):5329–39. doi: 10.1021/acsnano.2c08096 (PMC10061923; doi:10.1021/acsnano.2c08096)
Supplement: Supplementary file 1 — nn2c08096_si_001.pdf [file nn2c08096_si_001.pdf]

SUPPLEMENTARY INFORMATION

A high entropy oxide as high-activity electrocatalyst for water oxidation

Mohana V. Kante[1], Moritz L. Weber[2,3], Shu Ni[4], Iris C. G. van den Bosch[4], Emma van der Minne[4], Lisa Heymann[2], Lorenz J. Falling[3], Nicolas Gauquelin[5,6], Martina Tsvetanova[4], Daniel M. Cunha[4], Gertjan Koster[4], Felix Gunkel[2], Slavomír Nemšák[3,7], Horst Hahn[1,8], Leonardo Velasco Estrada[1,9,], Christoph Baeumer[2,4,]**

1 Institute of Nanotechnology, Karlsruhe Institute of Technology, Eggenstein-Leopoldshafen, 76344, Germany
2 Peter Gruenberg Institute and JARA-FIT, Forschungszentrum Juelich GmbH, Juelich, 52425, Germany
3 Advanced Light Source, Lawrence Berkeley National Laboratory, Berkeley, California 94720, USA
4 MESA+ Institute for Nanotechnology, University of Twente, Faculty of Science and Technology, Enschede, 7500 AE, Netherlands
5 Electron Microscopy for Materials Research (EMAT), Department of Physics, University of Antwerp, Antwerpen, BE-2020, Belgium,
6 NANOlab Center of Excellence, University of Antwerp, Antwerpen, BE-2020, Belgium
7 University of California Davis, Department of Physics and Astronomy, Davis, CA 95616, USA
8 The University of Oklahoma, Department of Chemical, Biological and Materials Engineering, Norman, OK 73019, USA
9 Universidad Nacional de Colombia sede de La Paz, La Paz, Cesar, 202010, Colombia

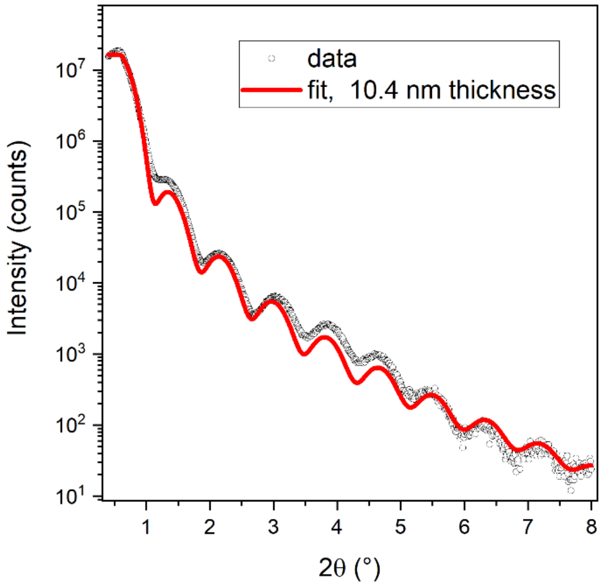

Supplementary Figure 1 XRR scan of $LaCr_{0.2}Mn_{0.2}Fe_{0.2}Co_{0.2}Ni_{0.2}O_3$ thin film.

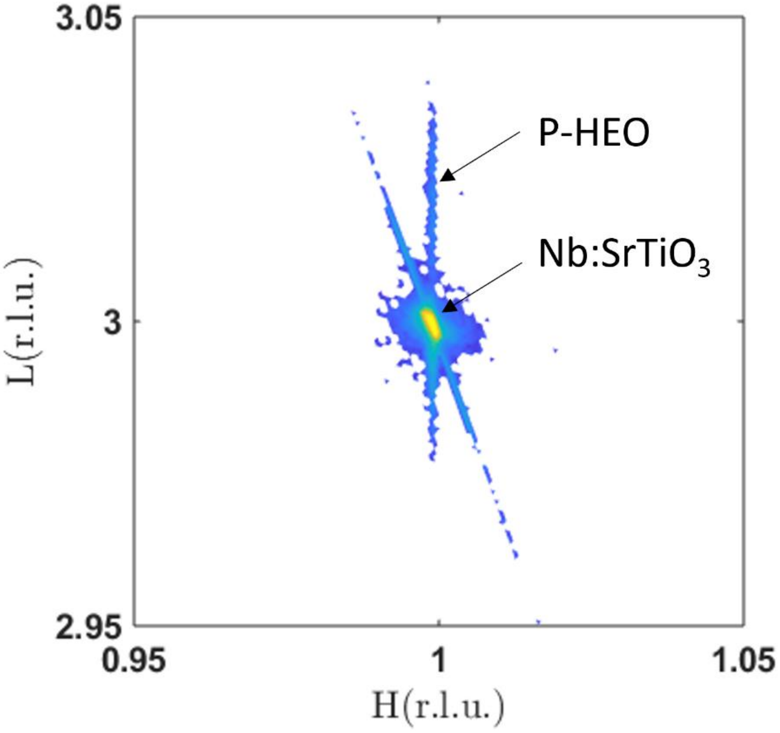

Supplementary Figure 2: Reciprocal space map around the (103) peak of a 11 nm P-HEO film on a $Nb:SrTiO_3$ substrate.

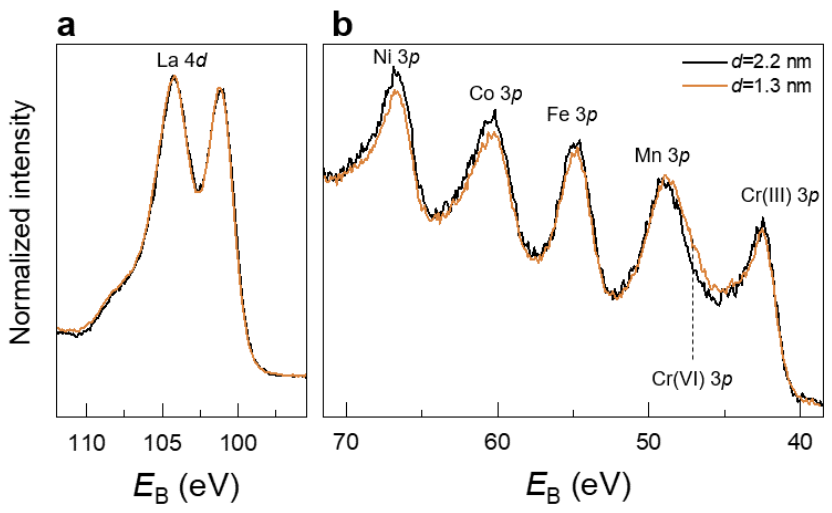

Supplementary Figure 3: XPS analysis with different mean escape depth *d* after in situ annealing in O_2. (a) La 4*d* peak. (b) TM 3*p* peaks. All data was normalized to the La 4*d* peak maximum, indicating that the film surface was slightly deficient in Ni, Co and Fe, indicating predominant (but incomplete) A-site termination. Mn and Cr are roughly stoichiometric.

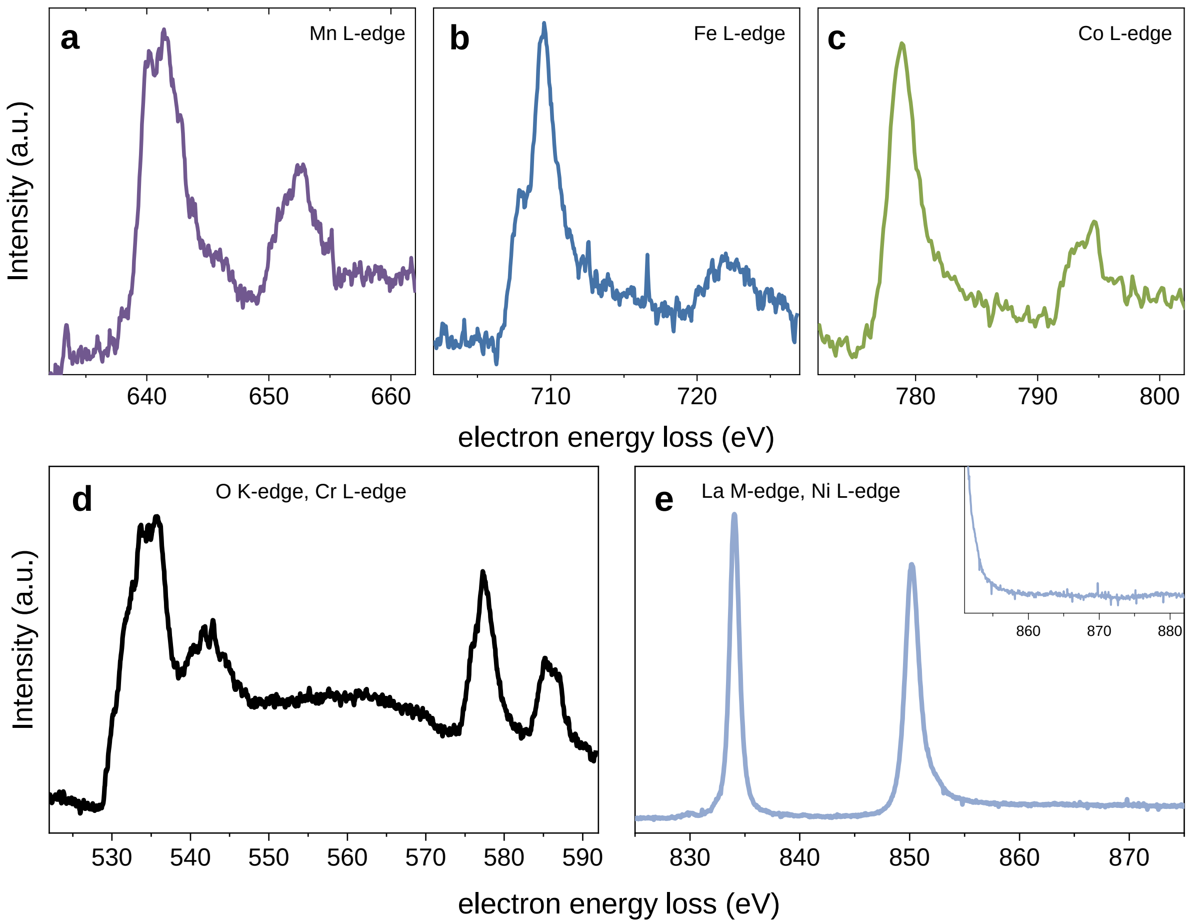

Supplementary Figure 4: EELS analysis. (a) Mn *L*-edge. (b) Fe *L*-edge. (c) Co *L*-edge. (d) Cr *L*-edge. (e) La *M*-edge, Ni *L*-edge. Inset: zoom to the Ni L-edge

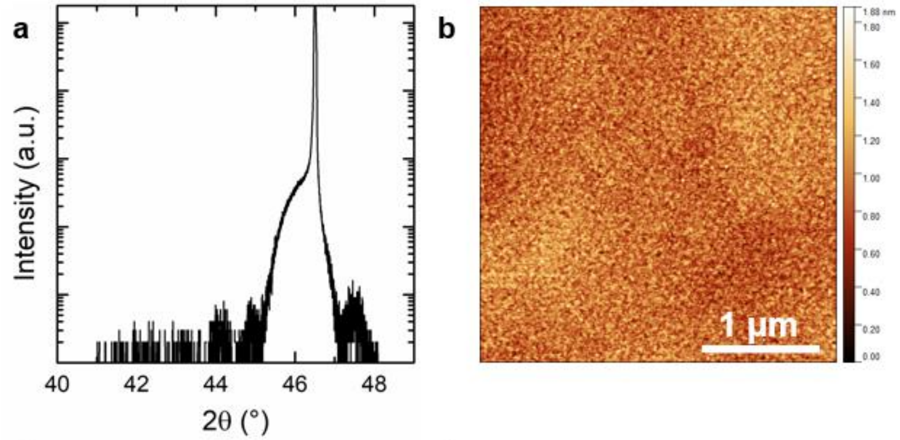

Fluence (J/cm²)	1.8
Pressure (mbar)	0.01
Temperature (°C)	750
Frequency (Hz)	2
# pulses	825
Growth Rate (p/ML)	27

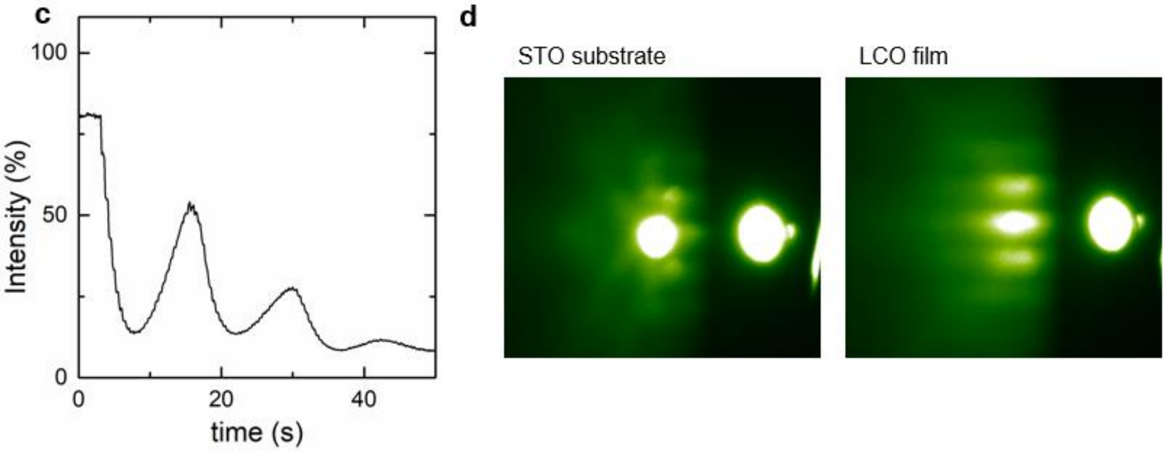

Supplementary Figure 5: Characterization of 12 nm as-prepared $LaCrO_3$ film. (a) HRXRD pattern (b) AFM morphology. (c) RHEED intensity oscillations during initial stage of growth. (d) RHEED pattern of the substrate and the film. Growth parameters are listed in the table.

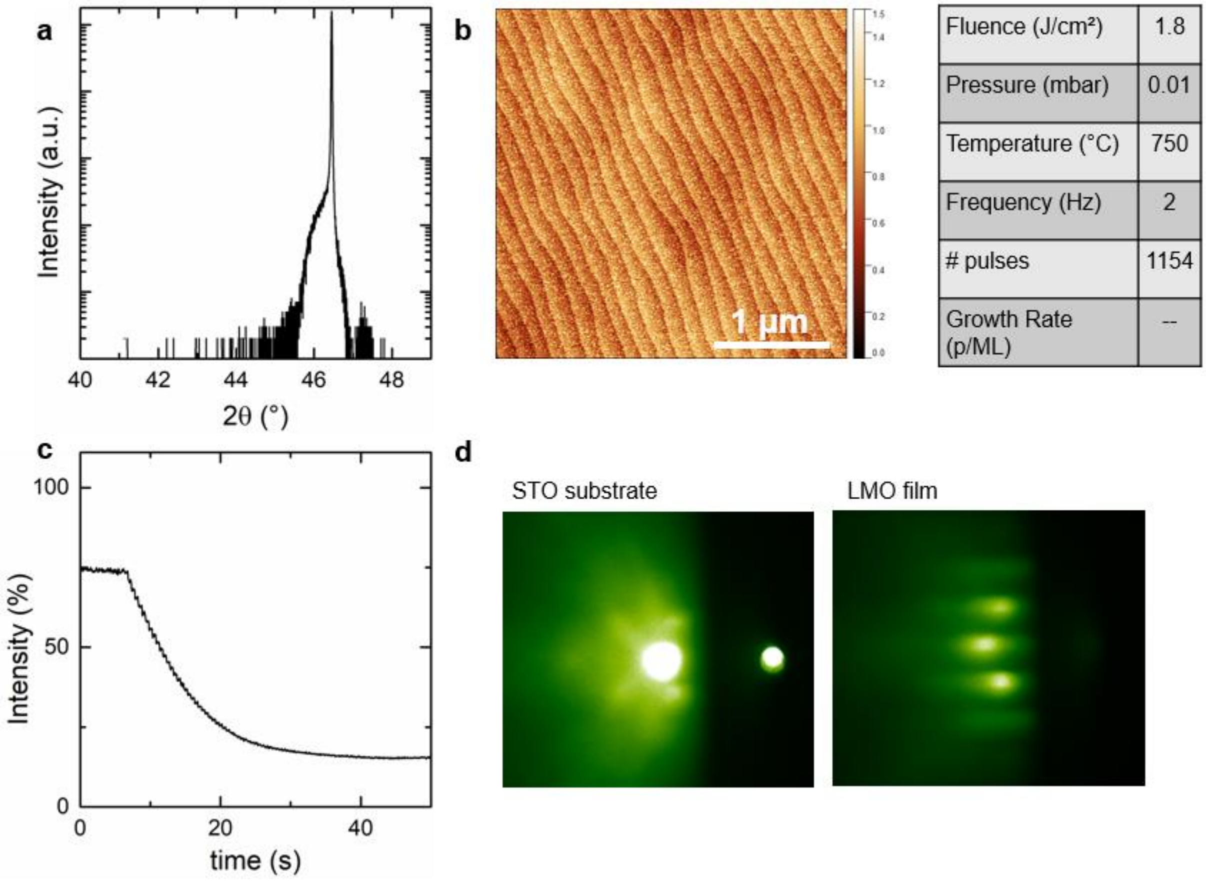

Fluence (J/cm²)	1.8
Pressure (mbar)	0.01
Temperature (°C)	750
Frequency (Hz)	2
# pulses	1154
Growth Rate (p/ML)	--

Supplementary Figure 6: Characterization of 12 nm as-prepared $LaMnO_3$ film. (a) HRXRD pattern (b) AFM morphology. (c) RHEED intensity oscillations during initial stage of growth. (d) RHEED pattern of the substrate and the film. Growth parameters are listed in the table.

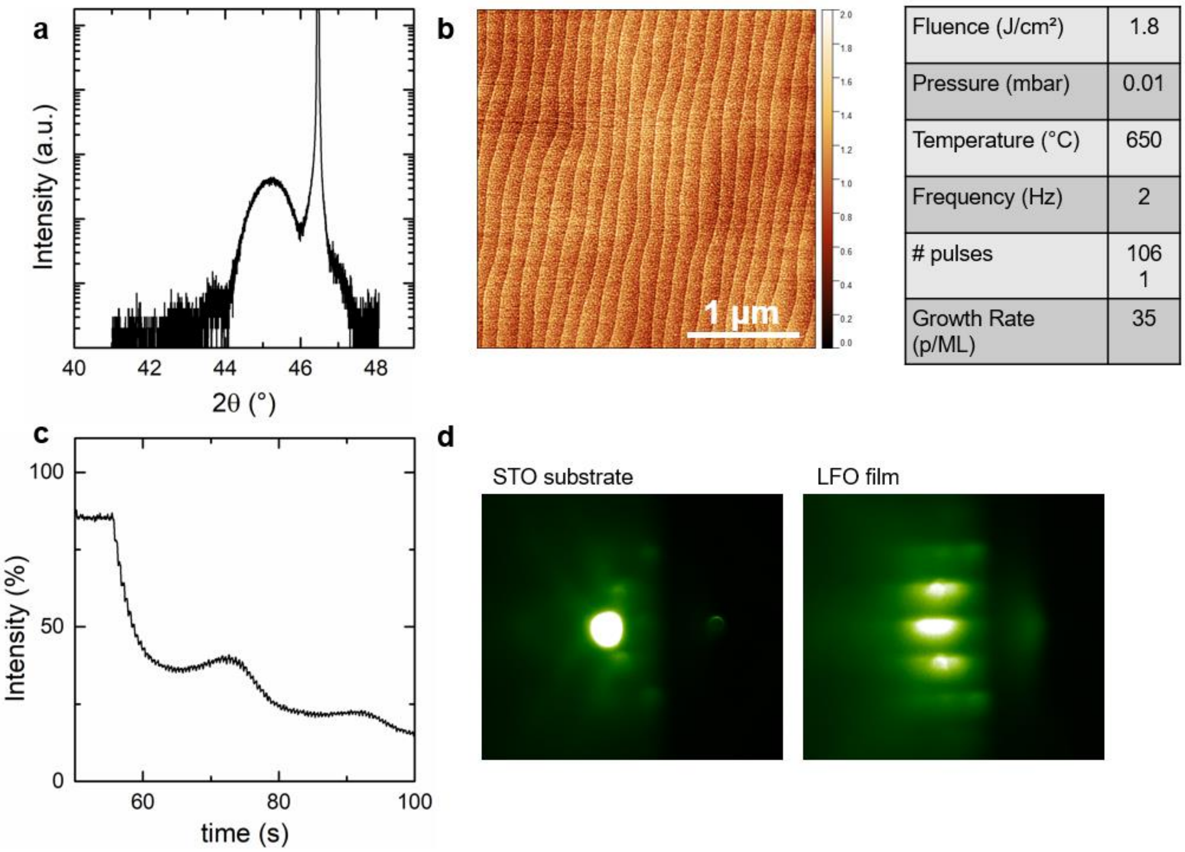

Fluence (J/cm²)	1.8
Pressure (mbar)	0.01
Temperature (°C)	650
Frequency (Hz)	2
# pulses	106<br>1
Growth Rate (p/ML)	35

Supplementary Figure 7: Characterization of 12 nm as-prepared $LaFeO_3$ film. (a) HRXRD pattern (b) AFM morphology. (c) RHEED intensity oscillations during initial stage of growth. (d) RHEED pattern of the substrate and the film. Growth parameters are listed in the table.

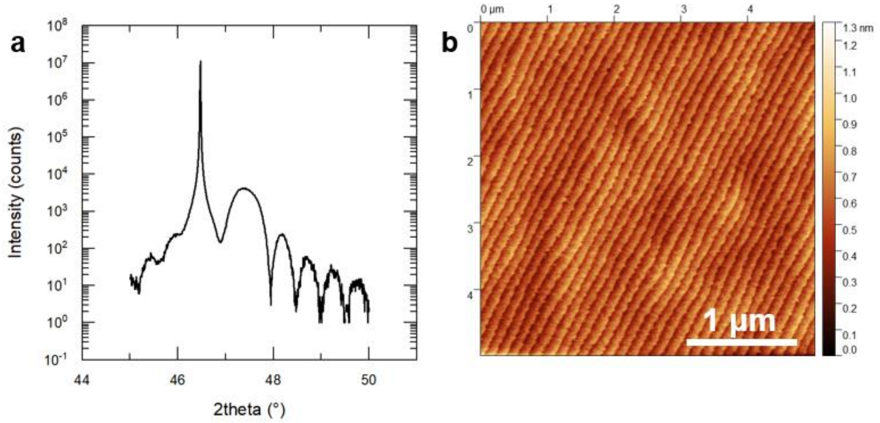

Fluence (J/cm²)	1.9
Pressure (mbar)	0.02
Temperature (°C)	700
Frequency (Hz)	5
# pulses	5375
Growth Rate (p/ML)	107.5

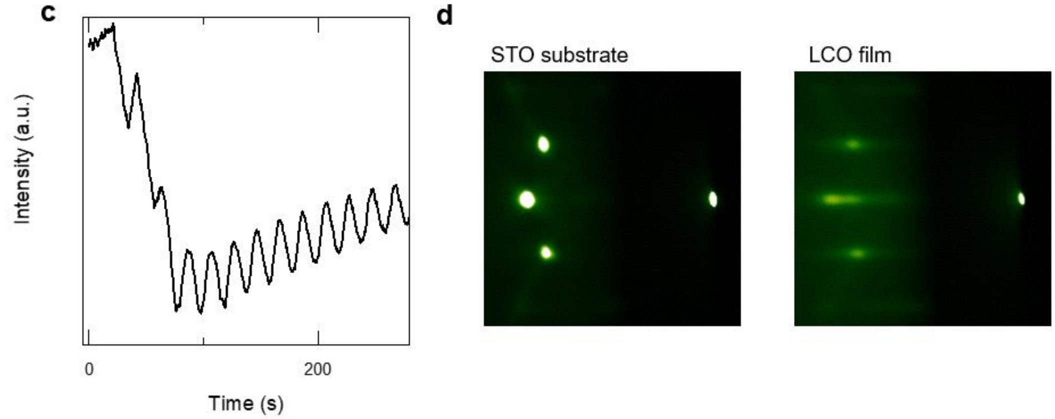

Supplementary Figure 8: Characterization of 20 nm as-prepared $LaCoO_3$ film. (a) HRXRD pattern (b) AFM morphology. (c) RHEED intensity oscillations during initial stage of growth. (d) RHEED pattern of the substrate and the film. Growth parameters are listed in the table.

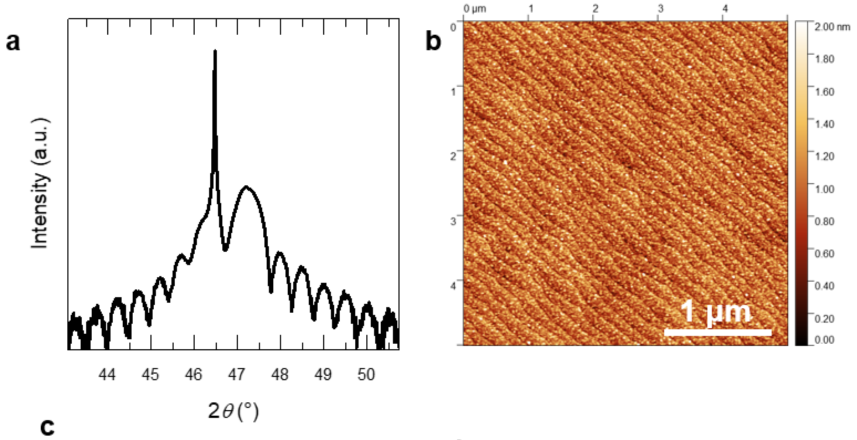

Fluence (J/cm²)	1.36
Pressure (mbar)	0.02
Temperature (°C)	700
Frequency (Hz)	5
# pulses	2000
Growth Rate (p/ML)	40

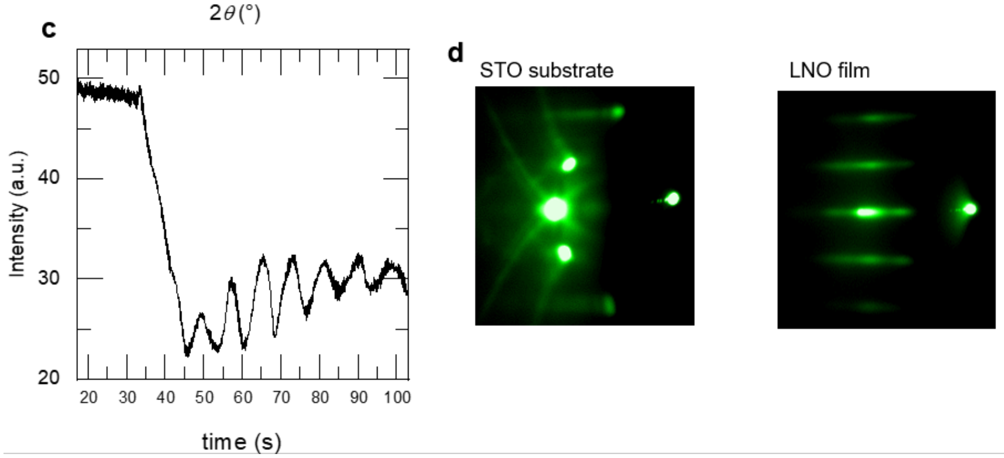

Supplementary Figure 9: Characterization of 20 nm as-prepared $LaNiO_3$ film. (a) HRXRD pattern (b) AFM morphology. (c) RHEED intensity oscillations during initial stage of growth. (d) RHEED pattern of the substrate and the film. Growth parameters are listed in the table. Electrochemical activity for 10 to 20 uc films was identical within experimental error.

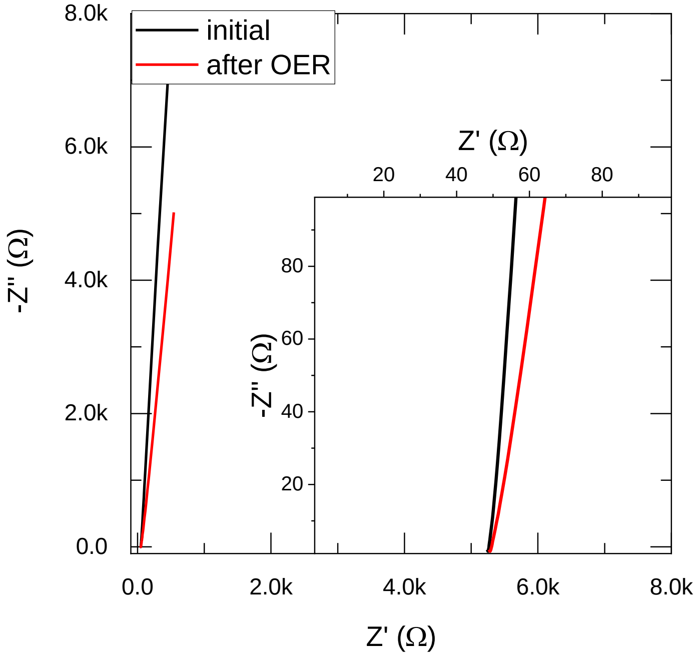

Supplementary Figure 10: Electrochemical impedance spectroscopy of a 12 nm P-HEO film before and after cyclic voltammetry in the OER regime.

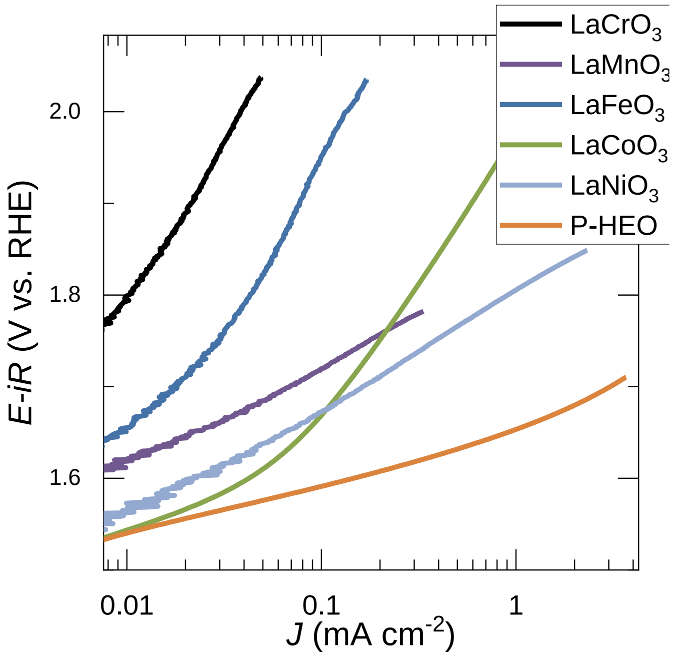

Supplementary Figure 11. Tafel analysis of the data in Figure 4 of the main text.

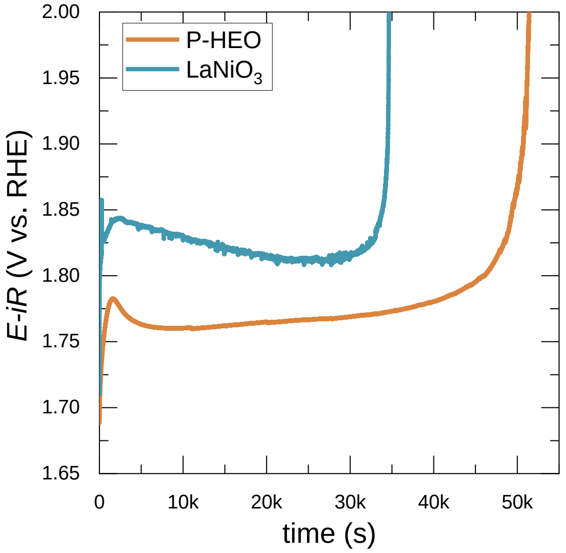

Supplementary Figure 12. Chronopotentiometry at 1 mA/cm^2 for P-HEO and $LaNiO_3$ (both La-terminated), indicating that the activity of P-HEO remain higher than for the parent compound. The P-HEO lifetime also appears to be enhanced, indicating that our P-HEOs exhibit sufficient stability to compare intrinsic OER activities.

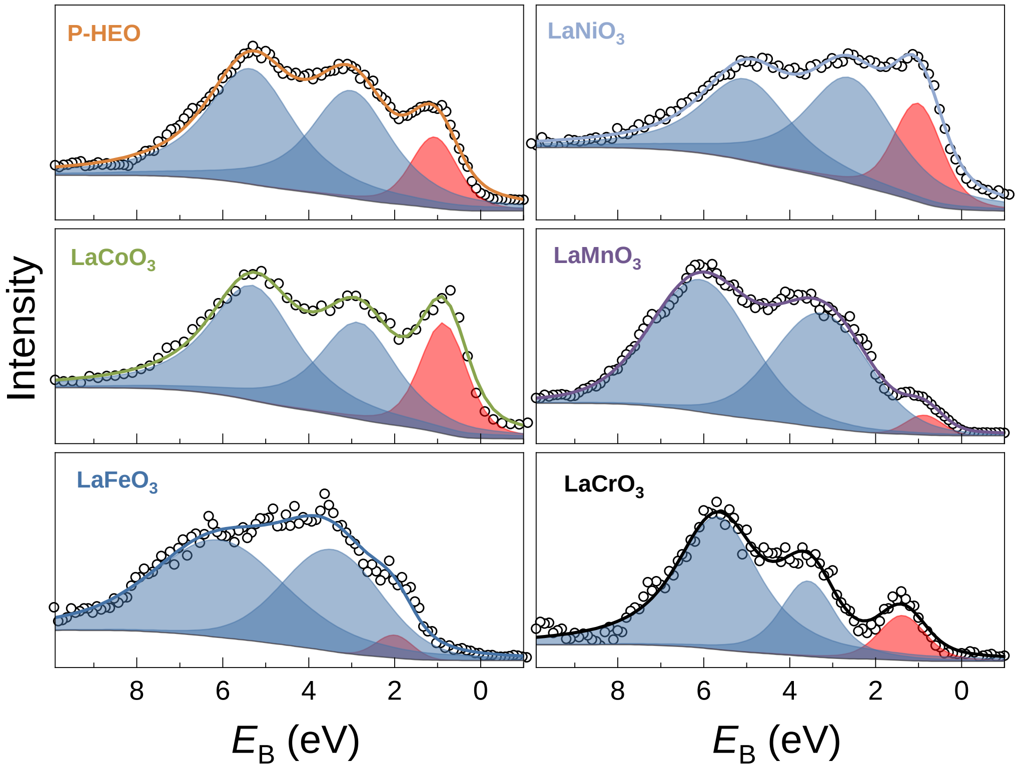

Supplementary Figure 13:Fitting for the valence band spectrum of P-HEO and the parent compounds. Oxygen O 2*p* states: peaks A and B, blue. TM 3*d* states: peak C, red.

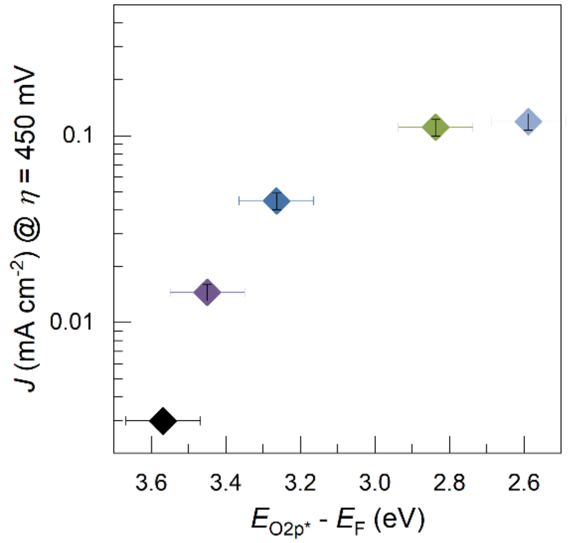

Supplementary Figure 14: Correlation between $E_{O_{2p}}$ and OER activity.

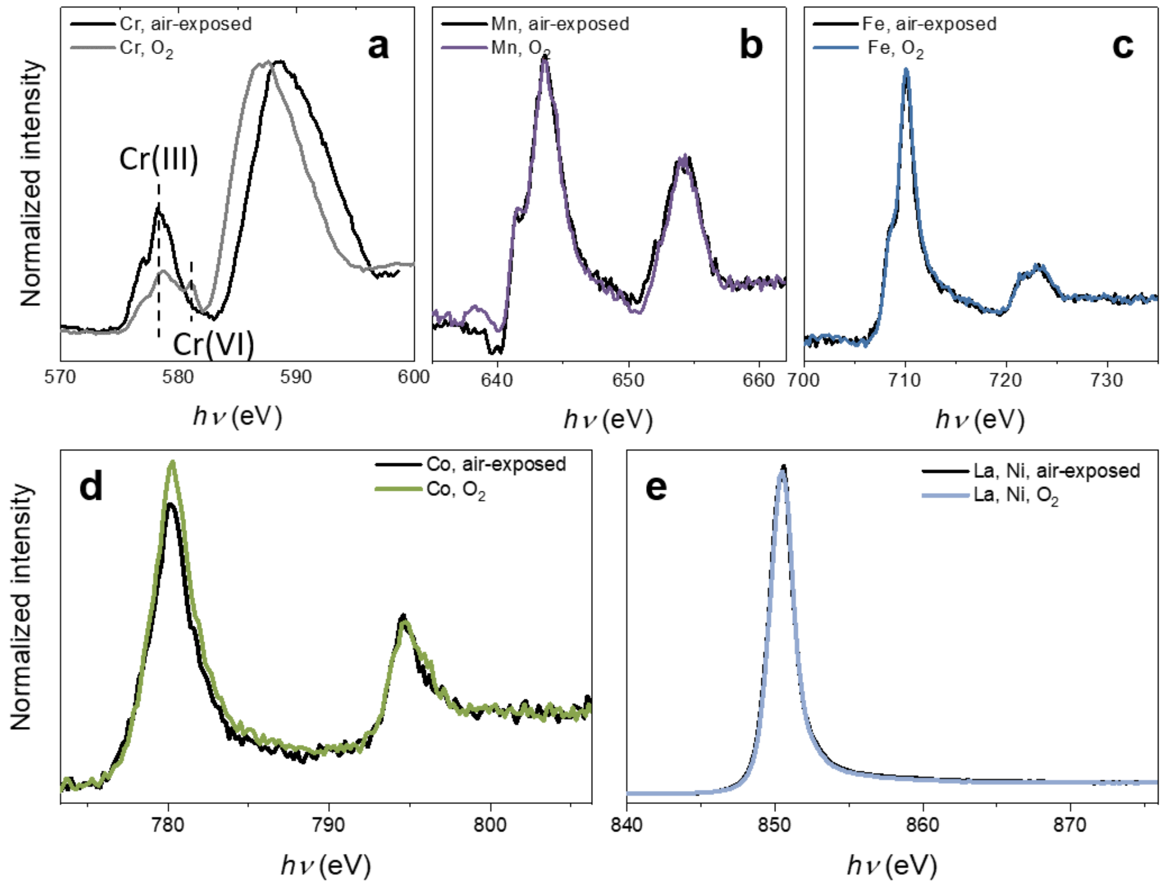

Supplementary Figure 15: Ambient pressure XAS analysis. (a) Cr *L*-edge. (b) Mn *L*-edge. (c) Fe *L*-edge. (d) Co *L*-edge. (e) La *M*-edge, Ni *L*-edge. Colored lines: measured in O_2 ambient. Black lines: measured after air-exposure. The white line sum rule indicates higher Co oxidation state after O_2 annealing.

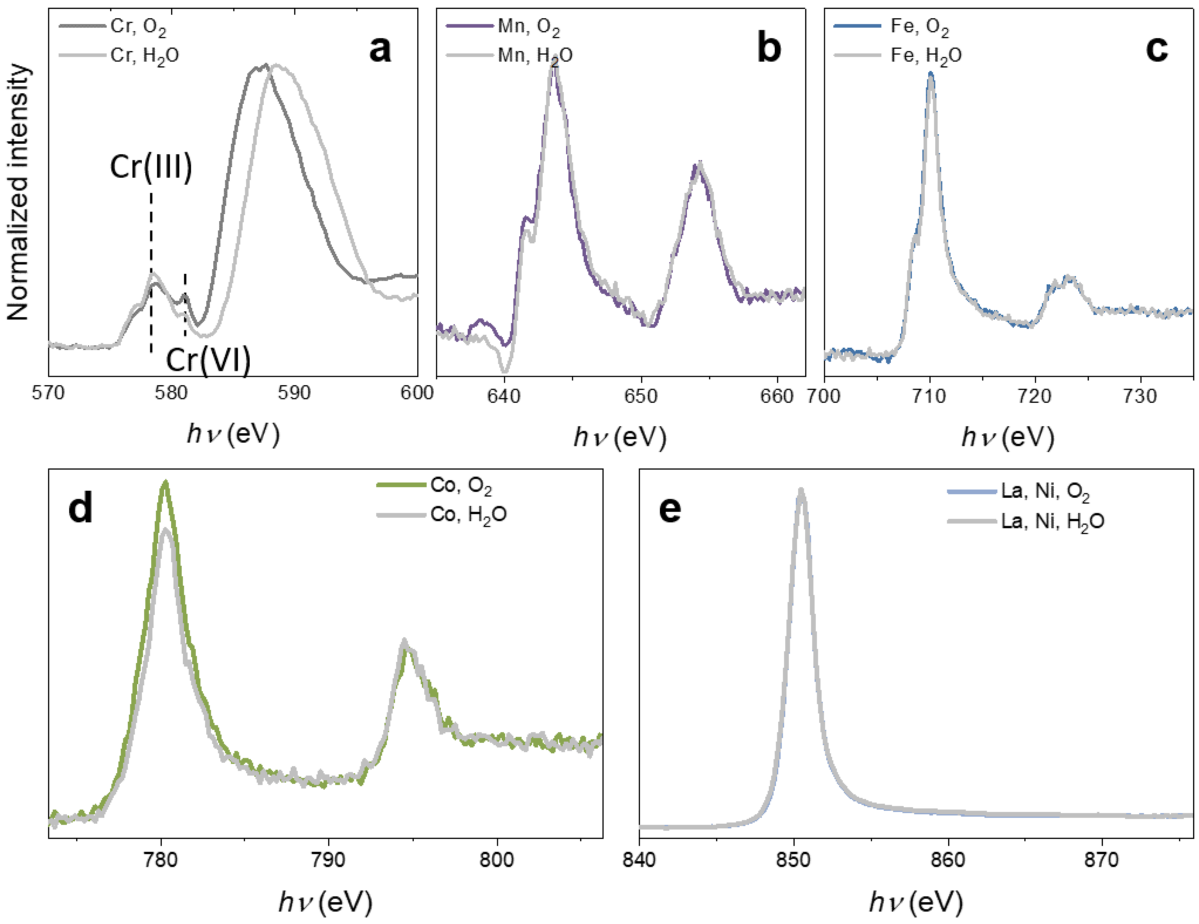

Supplementary Figure 16: Ambient pressure XAS analysis. (a) Cr *L*-edge. (b) Mn *L*-edge. (c) Fe *L*-edge. (d) Co *L*-edge. (e) La *M*-edge, Ni *L*-edge. Colored lines: measured in O_2 ambient Grey lines: measured in H_2O ambient at RT.
